# Editorial: Targeting the immunogenicity of cancer cells in anticancer therapies: from innate immunity to adaptive immune system implications

**DOI:** 10.3389/fimmu.2026.1876313

**Published:** 2026-05-22

**Authors:** Ana Carolina Martínez-Torres, José Correa-Basurto, Daniel Scott-Algara

**Affiliations:** 1 Laboratorio de Inmunología y Virología, Facultad de Ciencias Biológicas, Universidad Autónoma de Nuevo León, San Nicolás de los Garza, Mexico; 2 Laboratorio de Diseño y Desarrollo de Nuevos Fármacos e Innovación Biotecnológica, Sección de Estudios de Posgrado e Investigación (SEPI), Escuela Superior de Medicina, Instituto Politécnico Nacional, Ciudad de México, Mexico; 3 International Affair Department, Institut Pasteur, Paris, France

**Keywords:** anticancer therapies, cancer immunogenicity, immune system evasion, immunogenic cell death, immunotherapies, innate and adaptive immunity

One of the central challenges in cancer biology that hinders the development of effective anticancer therapies is the ability of cancer cells to evade immune detection. This immune escape is driven by multiple mechanisms, including the downregulation of antigen presentation, the establishment of an immunosuppressive tumor microenvironment, and inhibiting immune cell function, among others (1). Recent research has highlighted the complex interplay between cancer cells and the immune system, revealing how malignant tumor cells (MT) manipulate both innate and adaptive immune responses to favor their survival and progression (2, 3). Despite significant progress in understanding these complex biological processes, important gaps remain in the development of effective strategies to counteract immune evasion and promote cancer cell elimination by immune cells.

This Research Topic explored current advances in understanding and targeting the immunogenicity of cancer cells, with particular emphasis on the role of both innate and adaptive immunity. The eight contributions included reflect the broad diversity and complexity of the mechanisms involved in cancer cells–immune system interactions and provide therapeutic perspectives by exploring innovative strategies aimed at enhancing the immunogenicity of cancer cells and restoring immune recognition and MT elimination.

## Innate immune training and activation to potentiate cancer cell elimination

Innate immune cells play a pivotal role in initiating the immune response. They also potentiate the immune response to reinfection through an innate form of immunological memory known as trained immunity. The exploitation of trained immunity is a current area of intense research in the fields of vaccination, immunotherapies, infectious diseases, and cancer, among others. In this sense Chilibroste et al. investigated the capacity of *Salmonella* LVR01 to induce trained immunity and modulate antitumor responses. The authors found that the *in vivo* administration of *Salmonella* LVR01 can induce a trained immune response, as evidenced by increased cytokine secretion by bone marrow cells following secondary stimulation and reduced tumor growth in treated mice. In contrast, *in vitro* stimulation of *Salmonella* LVR01-pretreated human monocytes and murine myeloid cells resulted in reduced pro-inflammatory cytokine secretion. Other studies suggest that the ligand-receptor interaction could be an important factor in the differences between trained and tolerance responses (4), although the ligand used in the experiments reported herein was consistent. Also, the duration of the trained immunity induced by *Salmonella* LVR01 appears to differ from other trained immunity responses reported to persist for several months and associated with long-term epigenetic changes. These results suggest that the balance between trained immunity and its characteristics may depend on the cellular context and microenvironmental factors yet to be identified.

Drug resistance remains one of the principal limitations of chemotherapy against MT due to several mechanisms including the overexpression of P-glycoprotein (P-gp), an efflux pump encoded by ABCB1 in MT cells. The overexpression of P-gp has been linked to Toll-Like Receptor (TLR) stimulation, specifically, TLR2. For this reason, Álvarez-Carrasco et al. investigated the direct and paracrine effects of TLR2 stimulation on P-gp function in natural killer (NK) cells from pediatric patients with acute lymphoblastic leukemia (ALL). The authors examined the differential effects of TLR2/1 and TLR2/6 agonists to study functional activation and substrate-specific drug efflux, aiming to identify a strategy to protect immune effector cells during cancer therapy. Importantly, the functional activation of NK cells and the subsequent modulation of P-gp activity are strictly dependent on the specific TLR2 heterodimer involved. While TLR2/6 agonists primarily drive paracrine-dependent cytokine production, the TLR2/1 agonist PCSK triggers a robust direct cytotoxic response and selectively enhances methotrexate efflux. Importantly, this modulation does not come at the cost of effector function; rather, TLR2/1 stimulation significantly potentiates NK cell cytotoxicity against ALL cells while preserving effector viability. These findings propose a dual therapeutic benefit for TLR2/1 agonists in ALL by protecting NK cells from chemotherapy-induced suppression via P-gp potentiation, while simultaneously enhancing their antileukemic activity.

## Therapeutic strategies targeting immunogenic cell death

Immunogenic cell death (ICD) is a molecular mechanism by which the cell activates its own machinery to undergo self-destruction in a manner that is detectable by the immune system. Its induction can promote the release of danger-associated molecular patterns and tumor antigens, thereby enhancing antigen presentation and activating the adaptive immune system. This mechanism of cell death has an important therapeutic potential as it can render immunologically cold tumors into hot and immunotherapy-sensitive (5). In their review, Guo et al. examine the dual role of ferroptosis in digestive tract tumors, emphasizing its context-dependent capacity to modulate antitumor immunity. The authors describe how the tumor microenvironment can influence ferroptosis induction through factors such as hypoxia, cancer-associated fibroblasts, and immune cell infiltration. Conversely, ferroptosis can promote the induction of ICD; however, when dysregulated or excessive, it may lead to immunosuppressive effects. The authors propose that combining low-dose ferroptosis inducers with immunotherapies may maximize ICD-related immune activation and enhance therapeutic efficacy. The review addresses the dual impact of ferroptosis in both suppressing and stimulating immune responses, as well as its potential synergistic effects with immunotherapies, suggesting that precise regulation and combination with immunomodulatory strategies may enhance antitumor immunity.

Isaeva et al. address oncolytic virus (OVs) therapies, which selectively recognize cancer cells, replicate within them, and induce tumor cell destruction. Their ability to selectively target MT cells while simultaneously promoting antitumor immune responses makes OVs a promising strategy for targeted cancer therapy. The OVs-induced ICD can promote the cancer-immunity cycle (CIC) which activates anti-cancer immune responses. The authors describe how genetic engineering has enabled the development of OVs with enhanced tumor specificity and immunostimulatory properties, improving the safety and efficacy of virotherapy. Also, authors discuss current strategies aimed at increasing the MT specificity of OVs and improving their safety which could be possible due to the selective recognition of OVs-cancer cells mediated by macromolecule-macromolecule interactions. Authors summarize and functionally categorize different biochemical approaches focusing on virus engineering to activate the immune system able for immunotherapy purposes. Transduction-targeting methods and non-transduction modifications are extensively reviewed, along with the mechanisms of ICD and viral modifications that contribute to efficient cancer cell killing and the induction of cancer-specific immunity. Finally, OVs are proposed as promising therapeutic tools for the next generation of cancer immunotherapy.

## Therapeutic advances and challenges in cancer immunotherapy

Cancer immunotherapy comprises a broad spectrum of therapeutic strategies that differ in both mechanism and clinical application, with therapeutic efficacy largely depending on tumor type and patient characteristics. In this context, the review by Aman et al. provides a comprehensive overview of current immunotherapeutic strategies, highlighting both their major advances and current limitations. The therapies discussed include immune checkpoint blockade, adoptive cell therapies, cancer vaccines, cytokine-based therapies, oncolytic virus therapies, and combinatorial approaches. The authors also address the principal challenges associated with these treatments, including therapeutic resistance, toxicity, manufacturing complexity, and interpatient heterogeneity, all of which limit clinical efficacy. To overcome these difficulties, the development of more personalized and integrative therapeutic strategies is necessary to enhance both adaptive and innate antitumor immune responses.

In a more specific context, Silihe Kamga and Fiering review the potential of intratumoral immunotherapy (ITIT) administered prior to cancer surgery as a promising therapeutic strategy. The authors highlight that, despite significant advances in immunotherapy, only a limited proportion of patients currently benefit from these treatments. Thus, they propose that neoadjuvant immunotherapy, defined as the administration of immunotherapeutic agents before surgical tumor resection, may improve overall survival outcomes. This approach involves the local delivery of immunostimulatory agents to the tumor site prior to surgery, aiming to suppress immunosuppressive components of the tumor microenvironment while simultaneously activating immune responses. Once initiated, both innate and adaptive immune cells can target tumor antigens at primary and metastatic sites, potentially through systemic effects such as the abscopal response. Following tumor resection, circulating immune cells may continue to recognize residual tumor antigens, contributing to sustained tumor control and the development of long-term immunological memory. The authors also discuss emerging clinical evidence supporting the use of ITIT in the neoadjuvant setting, underscoring its potential to enhance antitumor immunity and improve clinical outcomes.

Katsuya et al. present a case of a rare phyllodes breast tumor with high tumor mutational burden (TMB) identified through comprehensive genomic profiling (CGP) and treated with pembrolizumab. Following several treatment cycles, clinical imaging revealed a partial response, as demonstrated by a reduction in lymph node involvement. However, despite continued pembrolizumab treatment, disease progression was eventually observed. As the authors indicate, this case report highlights the potential clinical utility of CGP testing in rare cancers. These findings are further supported by a recent study including a large cohort of 54,185 individuals with advanced common and rare solid tumors in Japan (6). In that study, biomarker analyses revealed that a TMB of at least 20 mutations per megabase predicts improved outcomes across tumor types, regardless of microsatellite instability status, in high TMB patients receiving pembrolizumab. Collectively, these findings highlight the real-world clinical utility of CGP and its variability across tumor types.

Finally, Rodríguez-Aguillón et al. examine how chronic comorbidities influence cancer immunoediting and therapeutic responses, particularly in the context of immunotherapy. The interplay between cancer, the immune system, and chronic comorbidities underscores the complexity of translating immunological insights into clinical benefit. Key features of these comorbidities, including systemic low-grade inflammation, metabolic dysregulation, immune dysfunction, and the establishment of tolerogenic tissue microenvironments, represent major barriers to effective antitumor immunity. The authors highlight that conditions such as obesity, type 2 diabetes mellitus, non-alcoholic fatty liver disease, and cardiovascular disease, which are increasingly prevalent among cancer patients, remain underrepresented in cancer immunotherapy studies despite their significant impact on tumor–immune interactions and therapeutic outcomes. To address this gap, they propose the integration of comorbidities into experimental cancer models as a critical step toward improving immunosurveillance, limiting tumor immune escape, and optimizing personalized immunotherapy strategies depending on the specific conditions of each patient.
